# Malignant pleural mesothelioma: diagnostic value of medical thoracoscopy and long-term prognostic analysis

**DOI:** 10.1186/s12890-018-0619-3

**Published:** 2018-04-03

**Authors:** Li-Li Xu, Yuan Yang, Zhen Wang, Xiao-Juan Wang, Zhao-Hui Tong, Huan-Zhong Shi

**Affiliations:** Department of Respiratory and Critical Care Medicine; Beijing Institute of Respiratory Medicine and Beijing Chaoyang Hospital, Capital Medical University, 8 Gongti Nanlu, Chaoyang District, Beijing, 100020 China

**Keywords:** Malignant pleural mesothelioma, Medical thoracoscopy, Prognosis

## Abstract

**Background:**

Malignant pleural mesothelioma (MPM) is marked by its difficult diagnosis and poor prognosis. Medical thoracoscopy (MT) is an effective and safe procedure for the diagnosis of exudative pleural effusions and many factors associated with poor prognosis of MPM. We conducted this study to investigate the value of MT for diagnosing of MPM and to identify prognostic factors for MPM patients.

**Methods:**

From July 2005 through June 2014, a total of 833 patients with undiagnosed pleural effusions underwent MT and pleural biopsies were taken. Clinical data of all patients with MPM were retrospectively analyzed, and those with complete follow-up data were analyzed for prognostic factors.

**Results:**

Eventually, MPM was the final diagnosis in 40 patients. Diagnostic efficiency of MT for MPM was 87.5%, since diagnosis of MPM failed to be established in 5 patients during the initial MT. Median survival was 17.1 mo (95% confidence interval: 13.6–20.7 mo). MT findings of pleural adhesion and plaques were adverse prognostic factors for MPM. In addition, old age, male gender, smoking history, histological type, poor staging, no treatment, low total protein level in pleural fluid, and computed tomographic findings such as pulmonary consolidation or infiltration, mediastinal lymphopathy, pulmonary mass or nodules, and pleural nodularity were also poor prognostic factors for MPM.

**Conclusions:**

MT is safe with a high positive rate in the diagnosis of MPM, and pleural adhesion and plaques seen under MT may be the adverse prognostic factors for MPM. Multiple clinical characteristics can affect the survival of MPM patients.

## Background

Malignant diffuse mesothelioma is a tumor arising from the mesothelial or submesothelial cells of the pleura, peritoneum, or pericardium. More than 80% of all mesothelioma originate in the pleura [1]. As malignant pleural mesothelioma (MPM) has been researched less than other primary neoplasms of the chest in the past few decades, it is of importance to study more about MPM, including its diagnosis and prognosis.

Clinical manifestations of mesothelioma are usually nonspecific. MPM is marked by its difficulty of diagnosis in the early stage. Because pleural effusion is usually the first clinical symptom of MPM, cytology of pleural effusion is often the first diagnostic examination to be performed. However, the accuracy of such an examination for the diagnosis of MPM is limited. The sensitivity of cytologic examination for a diagnosis of MPM was only 32% - 51.3% [2, 3]. Same problems are also observed in fine-needle biopsies or image-guided core needle biopsy. Medical thoracoscopy (MT) refers to the examination of the pleural space, and this procedure has been well documented to be highly sensitive of 87%–92.6% for diagnosing exudative pleural effusions with few complications which reported rates of 2% - 6% [4–6].

Once diagnosed as MPM, the median survival is short or only 4–16 mo [7–9]. Many factors associated with poor prognosis in patients with MPM, such as old age, poor performance status, advanced disease stage, thrombocytosis, chest pain, weight loss, asbestos exposure and long duration of symptoms [8, 10–13]. We conducted this study to investigate the usage of MT in the diagnosis of pleural effusion patients induced by MPM, and to identify general predictors of survival of MPM patients.

## Methods

### Patients

The study protocol was approved by the Institutional Review Boards for human studies of Beijing Chaoyang Hospital, China. Informed consents were not required as this was considered a review of clinical practice.

This study was a retrospective study. Between July 2005 and June 2014, a total of 833 patients with undiagnosed pleural effusions underwent MT and pleural biopsy was taken under direct visual control in the suspected areas such as pleural plaques and nodules, and systematically in several parts of the parietal pleura for mycobacterial, cytological, histological and immunohistochemical examination in our institution, and their detailed medical history, clinical presentation, laboratory examination results, and image data were recorded [6]. Only those data of pleural effusion patients with definite diagnosis of MPM were included in the current study, yielding 40 cases. Clinical stage was defined by the 7th TNM classification [14]. The initial MT followed by pathological and immunohistochemical analysis of pleural biopsy led to the definite diagnosis of MPM in 35 of 40 these patients. The remaining 5 MPE patients could not obtain the correct diagnose after the initial MT and the pleural biopsy only gave the result of nonspecific pleurisy. After 8 mo follow-up, 2 of the 5 patients was diagnosed as MPE by the second MT, one was diagnosed by percutaneous needle lung biopsy, one was diagnosed by open-long biopsy and one was diagnosed by liver-biopsy [15]. Before MT, all patients underwent the initial diagnostic workup, which included a detailed medical evaluation, CT scans, pleural fluid analyses, and/or closed pleural biopsy examination.

### Thoracoscopic procedures

MT was performed by chest physicians in our pulmonary procedural suite as described in our previous publications [6]. Pleural fluid and pleural biopsy samples obtained from each patient were analyzed. Cytological examination and biochemical parameters of pleural fluids and histopathological and immunohistochemical examination of pleural biopsies from all patients was performed. For pleural effusion, the effusion size of 300 ml - 500 ml was identified as small pleural effusion, the effusion size of 500 ml – 800 ml was identified as moderate pleural effusion, and the effusion size ≥800 ml was identified as large pleural effusion.

### Follow-up

We followed up individual patients until their death. During the follow-up, the following information was required from the patients or relatives every month by telephone or personal interview: 1) the demographic characteristics of patients; 2) the therapeutic regimen (surgical excision, any chemotherapy, radiation therapy, or pleurodesis) after diagnosed with MPM; 3) the time of death.

### Diagnostic criteria for MPM

Histopathology and immunohistochemistry review of all slides with at least one paraffin block representative pleural biopsy samples obtained through MT. The diagnosis of MPM was made by a panel of specialized pulmonary pathologists according to the current guideline [16]. MPM is divided into epithelioid, biphasic, and sarcomatoid subtypes on the basis of the predominant histomorphological growth pattern.

### Statistical analysis

All analyses were performed with SPSS software (version 19.0, SPSS Inc., Chicago, IL, USA). Data are presented as mean ± standard deviation (SD) or number with percentage. Descriptive statistical methods were used for data analysis.

We stratified patients by various factors including age, gender, smoking history, MT findings, histological type, staging, therapeutic regimen, TP of pleural fluid, LDH of pleural fluid and CT imagings. The rate of death was calculated for each level of the factor. MT findings, including pleural nodules, pleural hyperemia, pleural adhesion, pleural edema and pleural plaques, were examined to evaluate the relation with prognosis. The following other factors were also examined for the prognostic value: age, gender, smoking history, histological type, staging, treatment after diagnosed with MPM, laboratory levels of pleural fluid: total protein, lactate dehydrogenase and computed tomography (CT) imaging: pulmonary consolidation or infiltration, pulmonary atelectasis, mediastinal lymphopathy, pleural thickening, pulmonary mass or nodules, pleural nodularity.

Kaplan–Meier analyses were used and survival curves were plotted. Log rank test compared survival curves. Associations between possible prognostic variables and survival were estimated using Cox proportional hazards regression. All reported *P* values were two-sided and effects were considered significant if *P* <  0.05.

## Results

### Characteristics of patients with MPM

Between July 2005 and June 2014, 833 patients with undiagnosed pleural effusions successfully underwent MT, and pleural biopsy samples were obtained for diagnostic evaluation. Eventually, MPM was the final diagnosis in 40 patients. Patient characteristics are listed in Table 1. Thirty-five of 40 patients were diagnosed after pleural biopsy was taken by the initial MT. The remaining 5 patients could not get the correct diagnosis at the first time, we followed-up these patients and they were finally diagnosed as MPM [15].

**Table 1:** Characteristics of study subjects with confirmed malignant pleural mesothelioma after medical thoracoscopy (*n* = 40)

Variables	Values
Age, yr., median (range)	62 (40–78)
Sex, male, n (%)	20 (50.0)
Symptom	
Dyspnea	26 (65.0)
Chest pain	19 (47.5)
Cough	11 (27.5)
Fatigue	6 (15.0)
Fever	3 (7.5)
CT imaging, n (%)	
Pulmonary consolidation or infiltration	20 (50.0)
Pulmonary atelectasis	19 (47.5)
Pleural thickening	17 (42.5)
Mediastinal lymphopathy	11 (27.5)
Pulmonary mass or nodules	11 (27.5)
Pleural nodularity	7 (17.5)
Effusion site, n (%)	
Right	19 (47.5)
Left	16 (40.0)
Bilateral	4 (10.0)
Effusion size, n (%)	
Large	20 (50.0)
Moderate	9 (22.5)
Small	11 (27.5)
Thoracoscopic findings, n (%)	
Pleural nodules	32 (80.0)
Pleural hyperemia	19 (47.5)
Pleural adhesion	15 (37.5)
Pleural edema	5 (12.5)
Pleural plaques	5 (12.5)
Histology type	
Epithelioid	14 (35.0)
Sarcomatoid	9 (22.5)
Biphasic	5 (12.5)
Undifferentiated	12 (30.0)

As shown in Table 1, 20 patients were men and 20 were women, with their median age was 62 yrs., ranged from 40 yrs. to 78 yrs. The most common symptoms of MPM were dyspnea, chest pain, and cough. In 19 MPM patients, pleural fluid occurred on the right side, in 16 on the left, and in 4 both sides were affected. In either unilateral or bilateral effusion, the percentages of large, moderate, and small size of pleural effusions were 50%, 22.5%, and 27.5%, respectively. CT imaging revealed pulmonary consolidation or infiltration, pulmonary atelectasis, pleural thickening, mediastinal lymphopathy, pulmonary mass or nodules, and pleural nodularity.

### MT findings and pathological study

Under MT, one or more abnormalities could be observed on the surface of parietal or/and visceral pleura in all patients studied. We observed pleural nodules in 32 patients, pleural hyperemia in 19, pleural adhesion in 15, pleural edema in 5 and pleural plaques in 5 (Table 1).

After MT, histological and immunohistochemical examinations, 14 of 40 patients were diagnosed with epithelioid, 9 sarcomatoid, 5 biphasic and 12 undifferentiated (Table 1).

### Follow-up data of patients with MPM

Eventually, 33 of 40 patients were followed up until their death. The other 7 patients lost of follow-up. Median survival was 17.1 mo (95% confidence interval: 13.6–20.7 mo), range from 1.0 mo to 69.9 mo; 9 patients survived less than 1 yr., 18 survived less than 2 yrs., and 6 survived more than 2 yrs.

Most patients were in the late stages of disease (stage III or IV of the TNM staging system), with 9 patients were in stage II and no one was in stage I when diagnosis of MPM was established. Chemotherapy was performed in 21 of 33 patients; chemotherapy and radiotherapy were performed in 3 patients. Other 9 patients were not given any antitumor therapy.

### Assessment of prognostic factors for MPM

**Fig. 1:**
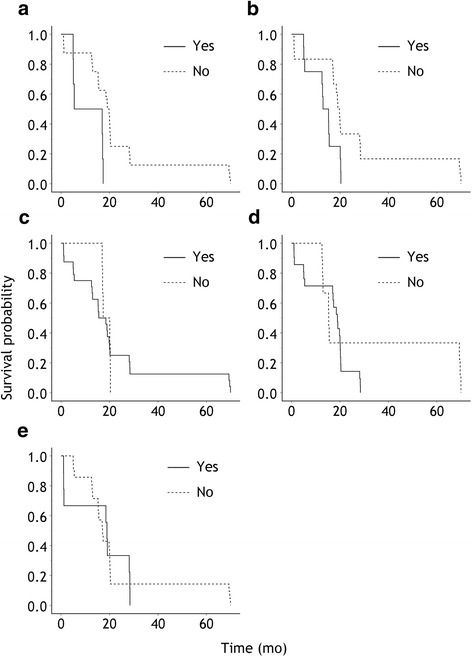
Kaplan–Meier survival curves for prognostic factors of findings under medical thoracoscopy (log-rank test). (**a**) Pleural adhesion (*P* = 0.010), (**b**) pleural plaques (*P* = 0.019), (**c**) pleural nodules (*P* = 0.985), (**d**) pleural hyperemia (*P* = 0.241), and (**e**) pleural edema (*P* = 0.814)

First of all, we focused on the prognostic value of MT findings, and noted that a statistically significant relation was detected on comparing overall survival and pleural adhesion and plaques with *P*-value of 0.016 and 0.023, respectively, with Hazard Ratio (95% Confidence Interval) of 0.277 (0.097–0.788) and 0.387 (0.170–0.879), respectively (Fig. 1).

Table 2 shows the univariate analyses of variables potentially impacting overall survival. Adverse prognostic factors included old age, male gender, smoking history, thoracoscopic findings such as pleural adhesion and plaques, histological type, poor staging and no treatment. Other adverse factors impacting prognosis like total protein level in pleural fluid and some CT findings were listed in Table 3.

**Table 2:** Association between clinic pathologic variables and survival

Prognostic factors	n	Median survival	Hazard Ratio	95% Confidence Interval	*P*
Age, yrs					
< 70	24	17.4			
≥ 70	9	5.1	1.81	1.165–2.813	0.008
Gender					
Male	12	13.0			
Female	21	20.0	0.346	0.142–0.844	0.020
Smoking history					
No smoking	21	20.0			
Smoking	12	5.4	6.752	2.418–18.855	< 0.001
Thoracoscopic findings					
Pleural nodules					
Yes	24	15.6			
No	6	17.4	1.009	0.399–2.554	0.985
Pleural hyperemia					
Yes	21	18.9			
No	9	15.3	0.573	0.223–1.468	0.246
Pleural adhesion					
Yes	6	5.4			
No	24	19.1	0.277	0.097–0.788	0.016
Pleural edema					
Yes	9	18.9			
No	21	17.1	0.908	0.404–2.038	0.814
Pleural plaques					
Yes	12	13.0			
No	18	19.1	0.387	0.170–0.879	0.023
Histological type					
Epithelioid	12	12.8			
Non-epithelioid	21	20.4	0.095	0.031–0.293	< 0.001
Staging					
I	0				
II	9	28.4			
III	15	17.1			
IV	9	3.0	21.042	6.156–71.923	< 0.001
Therapeutic regimen					
Chemotherapy	21	20.0			
Chemotherapy and radiotherapy	3	15.3			
No therapy	9	3.0	0.087	0.021–0.359	0.001

**Table 3:** Association between clinic variables and survival

Prognostic factors	n	Median survival	Hazard Ratio	95% Confidence Interval	*P*
TP of pleural fluid (g/L)					
< 40	12	3.2			
≥ 40	18	19.1	0.131	0.048–0.359	< 0.001
LDH of pleural fluid (IU/L)					
< 200	15	17.1			
≥ 200	15	15.3	1.276	0.591–2.753	0.535
CT imaging					
Pulmonary consolidation or infiltration					
Yes	9	3.0			
No	15	20.0	0.191	0.066–0.547	0.002
Pulmonary atelectasis					
Yes	18	13.0			
No	6	1.2	1.169	0.442–3.091	0.753
Mediastinal lymphopathy					
Yes	9	5.1			
No	15	20	0.230	0.080–0.661	0.006
Pleural thickening					
Yes	12	5.4			
No	12	13	0.693	0.288–1.666	0.412
Pulmonary mass or nodules					
Yes	9	5.1			
No	15	20.0	0.123	0.031–0.486	0.003
Pleural nodularity					
Yes	6	1.2			
No	18	13.0	0.340	0.120–0.964	0.043

*TP* total protein, *LDH* lactate dehydrogenase

**Fig. 2:**
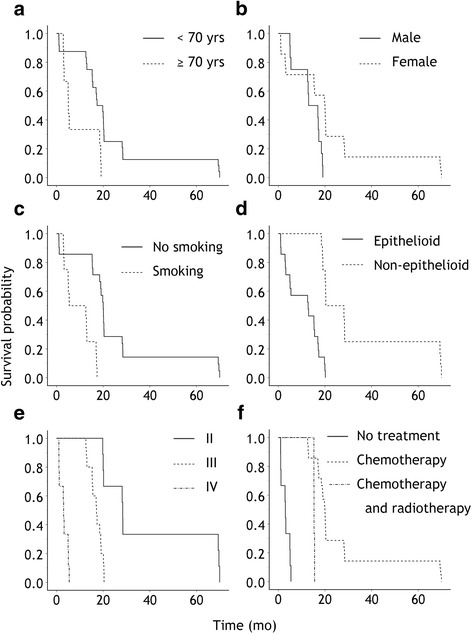
Kaplan–Meier survival curves for prognostic factors of general conditions (log-rank test). (**a**) Two age groups (*P* = 0.005), (**b**) two groups for gender (*P* = 0.015), (**c**) two groups for smoking history (*P* < 0.001), (**d**) two groups for histological types (*P* < 0.001), (**e**) three groups for staging (*P* < 0.001), (**f**) three groups for treatment (*P* < 0.001)

Figure 2 shows Kaplan–Meier survival curves subclassified 6 variables of general condition. According to our analysis, a statistically significant relation was detected on comparing overall survival and age, gender and smoking history with *P*-value of 0.008, 0.020, and <  0.001, respectively, and Hazard Ratio (95% Confidence Interval) of 1.81 (1.165–2.813), 0.346 (0.142–0.844) and 6.752 (2.418–18.855), respectively. A statistically relation was also detected on comparing overall survival and histological type, staging and treatment with *P*-value of < 0.001, < 0.001, and 0.001, respectively, and Hazard Ratio (95% Confidence Interval) of 0.095 (0.031–0.293), 21.042 (6.156–71.923) and 0.087 (0.021–0.359), respectively.

**Fig. 3:**
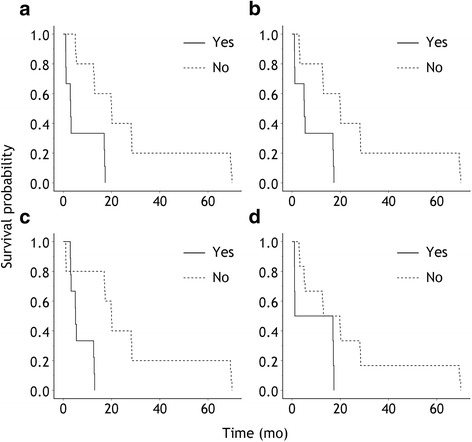
Kaplan–Meier survival curves for prognostic factors of findings on computed tomography (log-rank test). (**a**) Pulmonary consolidation or infiltration (*P* = 0.001), (**b**) mediastinal lymphopathy (*P* = 0.003), (**c**) pulmonary mass or nodules (*P* = 0.001), (**d**) pleural nodularity (*P* = 0.034)

Figure 3 shows the Kaplan–Meier survival curves that some CT imaging may also be the adverse prognostic factors, such as pulmonary consolidation or infiltration, mediastinal lymphopathy, pulmonary mass or nodules and pleural nodularity, with *P*-value of 0.002, 0.006, 0.003, and 0.043, respectively, and Hazard Ratio (95% Confidence Interval) of 0.191 (0.066–0.547), 0.230 (0.080–0.661), 0.123(0.031–0.486) and 0.340 (0.120–0.964), respectively.

## Discussion

The incidence of MPM has a growing tendency worldwide [17] and is likely to peak between the year 2015 and 2030 [18]. From July 2005 through June 2014, 833 patients with undiagnosed pleural effusions successfully underwent MT in our institute, 342 (41.1%) patients were confirmed to have malignant pleural effusion [6]. MPM (10.2%) is the second frequent cause of malignant pleural effusion. The most frequent cause is lung cancer (67.8%) [6].

Chest CT is commonly used in the preferred examination, which can display the surface of the whole pleura, the diaphragm and the lymph node [19]. The specificity of chest CT for identifying pleural tumors is about 88–95%, while the sensitivity is about 36–45% [20]. We noted in our study that MPM mainly appears as pulmonary consolidation or infiltration, as well pulmonary atelectasis on CT scan. Although it is still difficult in the tumor staging and distinguishing the diffuse pleural thickening from MPM [20], the images on CT scan can be used to guide subsequently biopsy and thus improve the diagnostic yield.

The diagnosis of MPM mainly relies on the pleural biopsy at present. Thoracoscopic pleural biopsy has become the most reliable method for the diagnosis of MPM for its comprehensive observation, accurately obtaining the tissue specimens, and the advantage of mini-invasive lesions [21]. In the present study, MPM was the final diagnosis in 40 patients with pleural effusion. Thirty-five of 40 patients were diagnosed by pleural biopsies which were taken during the initial MT, indicating that the diagnostic efficiency of MT for MPM is 87.5%. Our previous publication has reported that the overall diagnostic efficiency of MT for undiagnosed pleural effusions is 92.6% (771/833) [6]. The reason that MT has lower diagnostic efficiency for MPM may be multiple. Except the sampling error, this may, in part, be explained by the rapidly growing incidence of MPM, which can demonstrate various, misleading, histopathologic pitfalls, and the pleura is a common site for metastatic disease [22]. In addition, because neoplastic invasion of MPM occurs submesothelially, it may be difficult for a thoracoscopist to detect these areas on grossly normal appearing pleura [15]. To avoid this, biopsies should be taken as much as possible under the premise of no other side effects, especially in suspected areas such as pleural plaques and nodules. As a matter of fact, the diagnosis of MPM still requires a combination of clinical manifestations and other experimental results. MT as a real-time invasive procedure enables clinicians to improve tumor staging, particularly in the mediastinal region, by enabling the exact sampling point with adequate tissue for biopsy. Our current data support the consideration that MT is considered the best way for making diagnosis of MPM [23].

We analyzed survival in this retrospective cohort study of 40 patients diagnosed with MPM after MT in our hospital over a 10-yr period. Thirty-three patients with complete follow-up data were analyzed for variables potentially impacting overall survival. The median survival was 17.1 mo (95% confidence interval: 13.6–20.7 mo), range from 1.0 mo to 69.9 mo. Among the variables potentially affecting overall survival, first of all, pleural adhesion and plaques observed under MT are adverse prognostic factors for patients with MPM. These observations have not been mentioned in any one previous study. These results are reasonable since pleural biopsies were taken in the suspected areas under MT. This improves the authenticity of pleural biopsy and the diagnostic accuracy of histological types, which is related to the prognosis of MPM. We also noted that the other MT findings, such as pleural nodules, pleural hyperemia, and pleural edema, were not relative to prognosis of MPM. This could be explained that these findings can also be commonly found in many other pleural diseases.

Old age and male gender have been recognized as negative prognostic factors [12, 13, 24, 25], such findings are also seen in the present study. As is well-known that smoking was an important cause of lung cancer incidence and global mortality [18, 26], this result should not be beyond expectations. However, no previous study investigated the relationship between survival and smoking history, as most doctors pay more attentions on relationship between survival and asbestos exposure. We found that MPM patients with a smoking history had worse survival. We also found that low total protein level in pleural fluid and some CT images, such as pulmonary consolidation or infiltration, mediastinal lymphopathy, pulmonary mass or nodules, and pleural nodularity were important poor prognostic factors of MPM. These findings may provide implications for poor prognosis and more aggressive treatment.

Our data revealed longer survival for MPM patients with the epithelioid type and this result is in line with many previous studies [11, 12, 14, 21, 24, 27]. The presence of an inflammatory stromal response demonstrated an association with improved survival [28]. The histological type influences the choice of therapeutic strategy and epithelioid type typically resulting in more aggressive medical procedures. Staging describes the anatomical extent of the neoplasm [14]. A statistically significant relation was detected on comparing overall survival and advanced stage in our study, as mediastinal nodal involvement has been recognized as a critical component of staging, with a detrimental effect on survival [29–32]. The fact that no patient was diagnosed in stage I and only 9 patients was diagnosed in stage II revealed the difficulty of the early diagnosis of MPM.

Our patients with no tumor treatment had worse survival than patients with chemotherapy or chemotherapy plus radiotherapy. The association between no treatment and a poor prognosis might merely because that no treatment was given only when patients were in advanced staging and poor overall condition that cannot support any treatment. None of the 33 patients were performed with surgical treatment, so the relationship between overall survival and surgical treatment cannot be analyzed.

Our study had some limitations. First, the study sample size was not large, with only 40 MPM patients diagnosed after MT were included in our study, and only 33 of 40 patients who provided follow-up data were analyzed for prognosis. Using a small sample increases the chance of assuming as true a false premise. Thus, chances are that the patients with proposed poor prognostic factors have no shorter lifetime compared to patients without these factors. Second, we could only analyzed the data from MPM patients who underwent MT procedure, and no data from the other control groups, such as the patients with cancers and benign pleural diseases, were available, it was therefore not possible to calculate the sensitivity and specificity of MT in diagnosing MPM. Third, none of 33 patients underwent surgery, thus the relationship between surgical intervention and overall survival cannot be analyzed. In addition, there is a potential selection bias to this retrospective study. Therefore, more prospective studies with a larger cohort of subjects are needed to support the present findings.

## Conclusions

In summary, in view of difficulty of diagnosis and poor prognosis, the course of MPM remains aggressive and unfavorable. MT is safe with a high positive rate in the diagnosis of MPM, and pleural adhesion and plaques seen under MT may probably be the adverse prognostic factors of MPM. In addition, old age, male gender, smoking history, histological type, poor staging, no treatment, low total protein level in pleural fluid, and CT findings such as pulmonary consolidation or infiltration, mediastinal lymphopathy, pulmonary mass or nodules, and pleural nodularity may also be the poor prognostic factors for MPM. More prospective studies with a larger cohort of subjects are needed in the future to find the accurate prognostic factors for MPM.

## Abbreviations

MPM: Malignant pleural mesothelioma
MT: Medical thoracoscopy
SD: standard deviation
